# Investigating soluble CD40 ligand as a prognostic factor among acute coronary syndromes patients: A multi-center prospective case-control study

**DOI:** 10.1097/MD.0000000000039891

**Published:** 2024-09-27

**Authors:** Reham Nofal, Nafiza Martini, Majd Hanna, Imad-Addin Almasri, Ahmad Rasheed Alsaadi, Tahani Ali

**Affiliations:** a Faculty of Medicine, Damascus University, Damascus, Syria; b Medical Research Department, Stemosis for Scientific Research, Damascus, Syria; c Faculty of Economics, Statistics Department, Damascus University, Damascus, Syria.

**Keywords:** ACS, acute coronary syndrome, prognostic factor, prospective study, sCD40L

## Abstract

Soluble CD40 ligand (sCD40L) is a protein that plays a crucial role in the inflammatory response associated with the development and progression of acute coronary syndrome (ACS). Recent studies have suggested that sCD40L may be a useful prognostic factor for ACS, but the data are conflicting. This study aimed to investigate the potential of sCD40L as a prognostic marker among ACS patients and provide valuable insights for clinical practice. To our knowledge, this is the first study of its type in the Arabic World. A multi-center prospective case-control study was conducted in Damascus, Syria, involving 158 participants with different ACS subtypes (STEMI, NSTEMI, UA) and a control group of healthy individuals. Sociodemographic data, medical history, and sCD40L levels were collected. The predictive ability of sCD40L for ACS, STEMI, NSTEMI, and UA was assessed using receiver operating characteristic (ROC) curves. Statistical analysis was performed using IBM SPSS version 25. The study included 58 STEMI, 33 NSTEMI, 36 UA patients, and 30 healthy individuals. The mean age of participants was 55 years (SD 10.7 years). Analysis of sCD40L levels revealed significantly higher concentrations in ACS patients compared to the control group (*P* < .001). ROC curve analysis demonstrated that sCD40L had a significant predictive ability for ACS, STEMI, and NSTEMI (*P* < .05), while its predictive value for UA was not statistically significant. This study provides evidence supporting the potential of sCD40L as a prognostic factor in ACS. The elevated levels of sCD40L observed in these subtypes indicate its potential usefulness in risk stratification and predicting adverse cardiovascular events. Further investigations are warranted to establish standardized sCD40L cutoff values and evaluate its clinical implications in the management of ACS patients.

## 1. Introduction

Soluble CD40 ligand (sCD40L) is a protein that plays a crucial role in the inflammatory response associated with the development and progression of acute coronary syndrome (ACS).^[1]^ ACS is a group of conditions that includes unstable angina, non-ST-segment elevation myocardial infarction (NSTEMI), and ST-segment elevation myocardial infarction (STEMI).^[1]^ Soluble CD40L is generated through the cleavage of CD40L, which is a transmembrane glycoprotein belonging to the tumor necrosis factor superfamily that is expressed on activated T cells, platelets, endothelial cells, smooth muscle cells, and macrophages. The release of sCD40L into the circulation has been shown to be associated with the activation of the immune system and the development of atherosclerosis, which is a major risk factor for ACS.^[2]^ Recent studies have suggested that sCD40L may be a useful prognostic factor for ACS, patients with high levels of sCD40L had a higher risk of death, myocardial infarction, and revascularization compared to those with low levels of sCD40L.^[1]^ However, data from literature are conflicting, and the prognostic value of sCD40L in patients with ACS remains uncertain. Therefore, in the present study, we aimed to provide further evidence on the potential role of sCD40L as a prognostic factor in clinical practice among ACS patients.

## 2. Methods and materials

### 2.1. Study design and participants

This multi-center prospective case-control was conducted for approximately 1 year from January 2022 to January 2023 and was carried out at the cardiovascular department of 3 hospitals in Damascus, Syria: AL Mowasat University Hospital, Al Assad University Hospital, and Al Bassel Heart Surgery Hospital. The study population consisted of patients aged 18 years or older who presented to the emergency department or were admitted to the intensive care unit (ICU) with a diagnosis of acute coronary syndrome (ACS), including STEMI, NSTEMI, and Unstable Angina, as confirmed by history, clinical examination, laboratory tests, and Electrocardiography (ECG) by expert cardiologists.

However, certain patients were excluded from the study, including those who were discharged within 72 hours of admission to the ICU, had chronic coronary syndrome, had ECG showed Wellens/de Winter signs or new onset of Left/right bundle branch block. Additionally, patients with new coronary artery bypass, hematological/platelet disorders, autoimmune diseases including systematic lupus, liver failure, tumors and previous exposure to chemoradiotherapy, and those with a history of renal transplantation with immune rejection were also excluded from the study. In addition, healthy people were also recruited to compare between them and the 3 patients group (STEMI, NONSTEMI, Unstable angina). Additionally, a group of healthy individuals (Control group) were also recruited to participate in this study and only those who are nonsmokers with no chronic disease or cardia problems or previous usage of anticoagulant were eligible to participate as control group.

### 2.2. Data collection

In the course of this study, various pieces of information were collected. The following sociodemographic and medical history information was recorded: age, gender, smoking habits (smoker or nonsmoker), chronic diseases (diabetes, hypertension), previous myocardial infarction, family history of heart disease, use of anticoagulants or streptokinase, and history of cardiac catheterization or coronary artery bypass graft surgery (CABG). Electrocardiograms (ECGs) were performed and data about its findings regarding ST and T wave was recorded. Additionally, laboratory tests values of troponin, creatine kinase, creatine kinase-myocardial band and other values were conducted and recorded.

The final diagnosis of each case was determined based on clinical findings such as severe chest pain, sweating, and nausea, as well as ECGs changes and elevated cardiac biomarkers. Upon that, they were classified as ST-elevation myocardial infarction (STEMI), non ST-elevation myocardial infarction (NSTEMI), or unstable angina (UA). However, all patients underwent a cardiac catheterization to confirm artery stenosis.

#### 2.2.1. Definitions

The study defined STEMI patients as those who had biomarkers of myocardial necrosis, which included a typical rise and gradual fall of troponin or a more rapid rise and fall of creatine kinase MB. Additionally, STEMI patients had to meet at least one of the following criteria: a history of ischemic-type chest pain, the development of pathological Q waves on the electrocardiogram (ECG), or ECG changes indicative of ischemia.

On the other hand, patients were classified as having unstable angina (UA) or non-ST-segment elevation myocardial infarction (NSTEMI) if they experienced one of 3 possible types of chest pain: new onset angina that is severe and/or frequent (more than 3 episodes per day), accelerating angina, or angina at rest. These patients also had to have ECG changes, which included ST segment depression of more than 0.05 mV or T-wave inversion of more than 0.2 mV in the precordial leads. The difference between UA and NSTEMI was determined by the presence of elevated serum cardiac markers.

#### 2.2.2. Blood sampling and measurement of sCD40L and cardiac biomarkers

A sample of peripheral blood was drawn from patients hospitalized in the ICU, and the blood was collected into 2 tubes. The first tube contained EDTA to perform a complete blood count, which includes the measurement of white blood cells and red blood cells and other relevant values. The second tube did not contain any additives, allowing for the separation of serum. The samples were then stored at −80°C until further analysis which was done at the laboratory of University Hospital Obstetrics in Damascus. The serum was then used to determine the concentration of soluble CD40 ligand (sCD40L) through the enzyme-linked immunosorbent assay technique. This was accomplished using a human sCD40l kit from the RayBiotech Company, Peachtree Corners. Cardiac biomarkers were measured using standard laboratory techniques.

### 2.3. Sample size

The sample size (n) was determined by Cochran’s sample size formula with the assumption of 95.5% confidence level (*Z* = 2), *e* is the margin of error which is 10%, *p* is the (estimated) proportion of the population which has the attribute in question, and it equals 50% (or 0.5), and *q* is 1 − *p*


$$\begin{array}{l} n=\frac{z^{2}pq}{e^{2}}=\frac{{\left(2\right)}^{2}*0.5*0.5}{{\left(0.1\right)}^{2}}=100 \end{array}$$

The required sample size (n) for this study, applying the previous formula, is at least 100.

If we adopt a 5% margin of error. The sample size becomes 400.


$$\begin{array}{l} n=\frac{z^{2}pq}{e^{2}}=\frac{{\left(2\right)}^{2}*0.5*0.5}{{\left(0.05\right)}^{2}}=400 \end{array}$$

Therefore, with a confidence of 95.5 and an error ranging from 5% to 10%, a sample size ranging from 100 to 400 individuals should be required. We had sought to have relied on the extent of the error of 5%, so depending on the previous equation, we collected 158 participants.

### 2.4. Data analysis

The statistical analysis was performed using IBM SPSS version 25. Descriptive statistics such as means, standard deviations (SD), medians, frequencies, and percentages were calculated to summarize the data. To examine the relationships between variables, Pearson’s correlation test was employed. Furthermore, receiver operating characteristic (ROC) curves were constructed for sCD40L in ACS, STEMI, NSTEMI, and Unstable Angina cases to evaluate its predictive ability for developing ACS. All statistical tests were deemed significant at a 5% level of significance.

### 2.5. Ethical approval and confidentiality

The study obtained ethical approval from the institutional ethics committee of Damascus University, with a specific reference number (1787). The study was thoroughly explained to the patients, highlighting the voluntary nature of their participation and the ability to withdraw at any time without impacting their healthcare at the hospital. The patients’ anonymity was preserved, and their personal information, such as name, address, phone number, or email address, was not collected. Access to the data was restricted to the investigators only. Participants who agreed to participate provided written consent.

## 3. Results

### 3.1. Baseline characteristic of participants

This study included a total of 158 participants, with an average age of 55 years (standard deviation 10.7 years). The participants were divided into 58 with STEMI, 33 with NSTEMI, 36 with UA, and 30 healthy individuals. The majority of the participants were male (117 or 74.7%). Most of the patients were also smokers (92 out of 122). Since not smoking is one of the inclusion criteria of healthy individuals, none of the healthy group was smoker (Tables 1 and 2).

**Table 1:** Demographic and clinical characteristics of study participants.

Variables	Groups	Frequency	Percentage (%)
Gender	Male	118	74.7
	Female	40	25.3
ACS sub-groups	Normal	30	19.0
	STEMI	58	36.7
	Non-STEMI	34	21.5
	Unstable	36	22.8
Hypertension	No	59	46.1
	Yes	69	53.9
Diabetes	No	83	64.8
	Yes	45	35.2
History of cardiac infarction	No	78	60.9
	Yes	50	39.1
Previous cardiac catheterization	NO	85	66.4
	Yes	43	33.6
CABG	No	121	94.5
	Yes	7	5.5
Family history of heart disease	No	101	79.5
	Yes	26	20.5
Anticoagulants	No	79	61.7
	Yes	49	38.3
Streptokinase	NO	82	64.1
	Yes	46	35.9
Smoking status	Non smoker	60	39.5
	Smoker	92	60.5

ACS = acute coronary syndrome, CABG = coronary artery bypass grafting, STEMI = ST-elevation myocardial infarction.

**Table 2:** Summary statistics of clinical variables.

Variables	Frequency	Minimum	Maximum	Mean	Standard deviation
Age	158	27	80	55.16	10.70
sCD40L (ng)	158	0	263.74	42.69	53.18
sCD40L (pg)	158	1.29	263738.1	42690.07	53178.98
Troponin (0.04 ng/mL)	76	0	60	4.45	9.01
CK (0–170) U/L	90	31	5185	671.50	989.21
CK-MB (7–25) U/L	93	5	1450	100.49	177.34

CK = creatine kinase, CK-MB = creatine kinase muscle/brain, ng/mL = nanograms per milliliter, sCD40L = soluble CD40 ligand, U/L = units per liter.

In terms of chronic diseases, 69 out of 128 participants had hypertension (53.7%) and 45 out of 128 had diabetes (35.2%). Only 26 patients reported a family history of heart disease (20.5%). Nearly a third of the patients (50 out of 128) had a history of cardiac infarction, while the majority had not. Regarding previous interventional procedures, 43 patients (33.6%) had undergone cardiac catheterization, and only a small number had undergone CABG (7 out of 128 or 5.5%). Regarding treatment, out of 128 patients, 49 used anticoagulants (38.3%) and 46 used streptokinase (35.9%). The mean value of sCD40L was 42.69 ng with a standard deviation of 53.18 for all participants. Levels of cardiac markers in patients groups including troponin, CK, CK-MD are shown in Table 2.

### 3.2. The difference of sCD40L levels among ACS subgroups

Our research revealed a statistically significant difference in sCD40L levels across ACS subcategories (*P* value of 0). The average sCD40L concentration was notably higher in STEMI patients (68.48 ng) compared to NSTEMI, UA, and individuals without ACS, with mean levels of 29.26 ng, 24.69 ng, and 29.64 ng, respectively (Tables 3 and 4).

**Table 3:** Comparison of sCD40L levels across ACS subgroups.

Variables		ACS subgroups			
		Normal	STEMI	Non-STEMI	Unstable
sCD40L (ng)	MIN	0.22	1.9	0.02	0
	MAX	263.74	259.94	173.21	136.96
	Mean	29.64	68.48	29.26	24.69
	SD	51.92	61.43	42.37	29.30
	*P* value*	0			

*P* value indicates the statistical significance of the differences among groups, calculated using a 1-way ANOVA test.

ACS = acute coronary syndrome, sCD40L = soluble CD40 ligand, STEMI = ST-elevation myocardial infarction.

* Oneway ANOVA test.

**Table 4:** Mean differences in sCD40L levels among ACS subgroups.

ACS (I)	ACS (2)	Mean difference (I–J)	*P* value
Normal	STEMI	−38.83514*	.001
	Non-STEMI	0.38018	.976
	Unstable	4.95039	.688
STEMI	Non-STEMI	39.21531*	.000
	Unstable	43.78553*	.000
Non-STEMI	Unstable	4.57021	.702

Mean difference (I–J): The difference in mean sCD40L levels between the 2 ACS subgroups. *P* value indicates the statistical significance of the mean differences.

ACS = acute coronary syndrome, STEMI = ST-elevation myocardial infarction.

* The mean difference is significant at the .05 level.

Subsequent analyses were conducted to compare these subgroups, demonstrating significant differences in sCD40L concentrations between the healthy group and the STEMI group (*P* = .001), as well as between the STEMI group and both the NSTEMI (*P* = 0) and Unstable Angina (*P* = 0) groups.

### 3.3. Role of sCD40L as prognostic factor in predicting ACS, STEMI, NSTEMI, and UA

**Figure 1.**
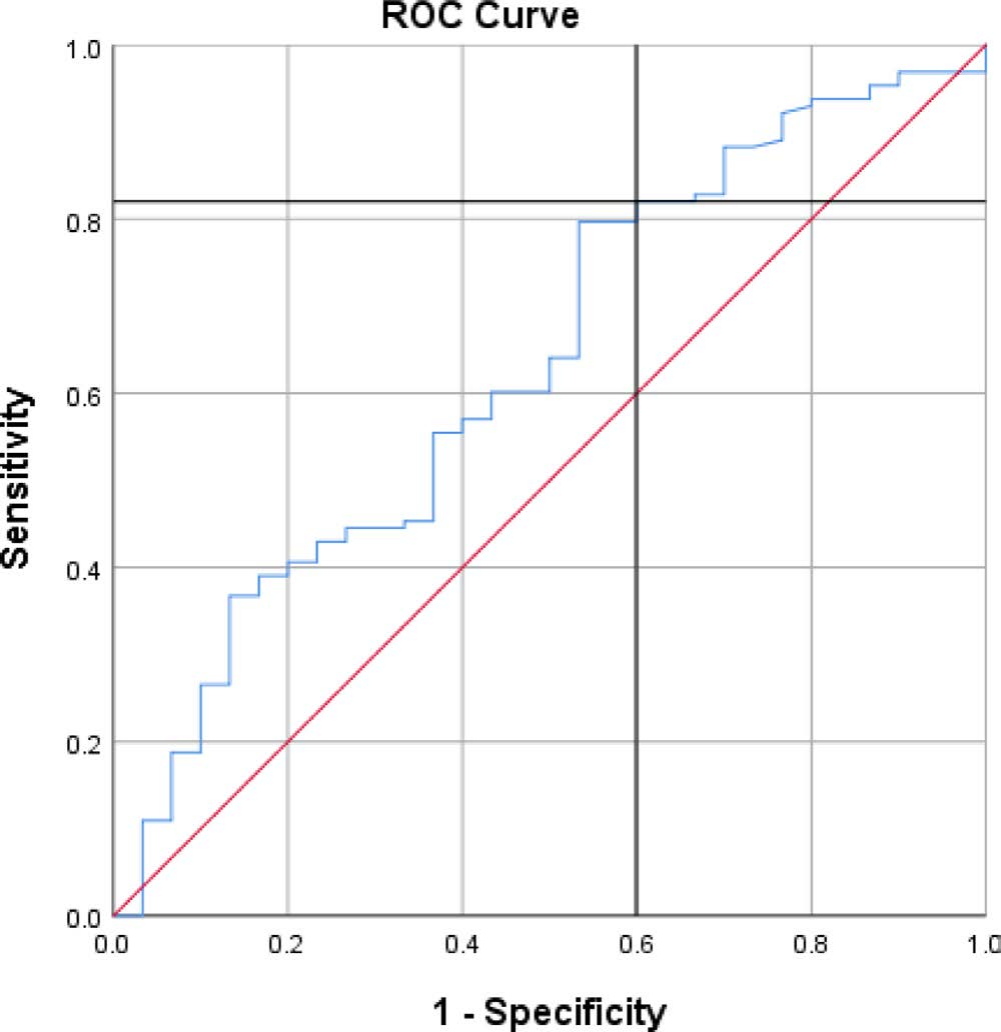
ROC curve for differentiation between ACS and healthy, AUC of 63.2% and a *P* value of .025 (sensitivity = 82%, specificity = 40%). ACS = acute coronary syndrome, AUC = area under curve, ROC = receiver operating characteristic.

**Figure 2.**
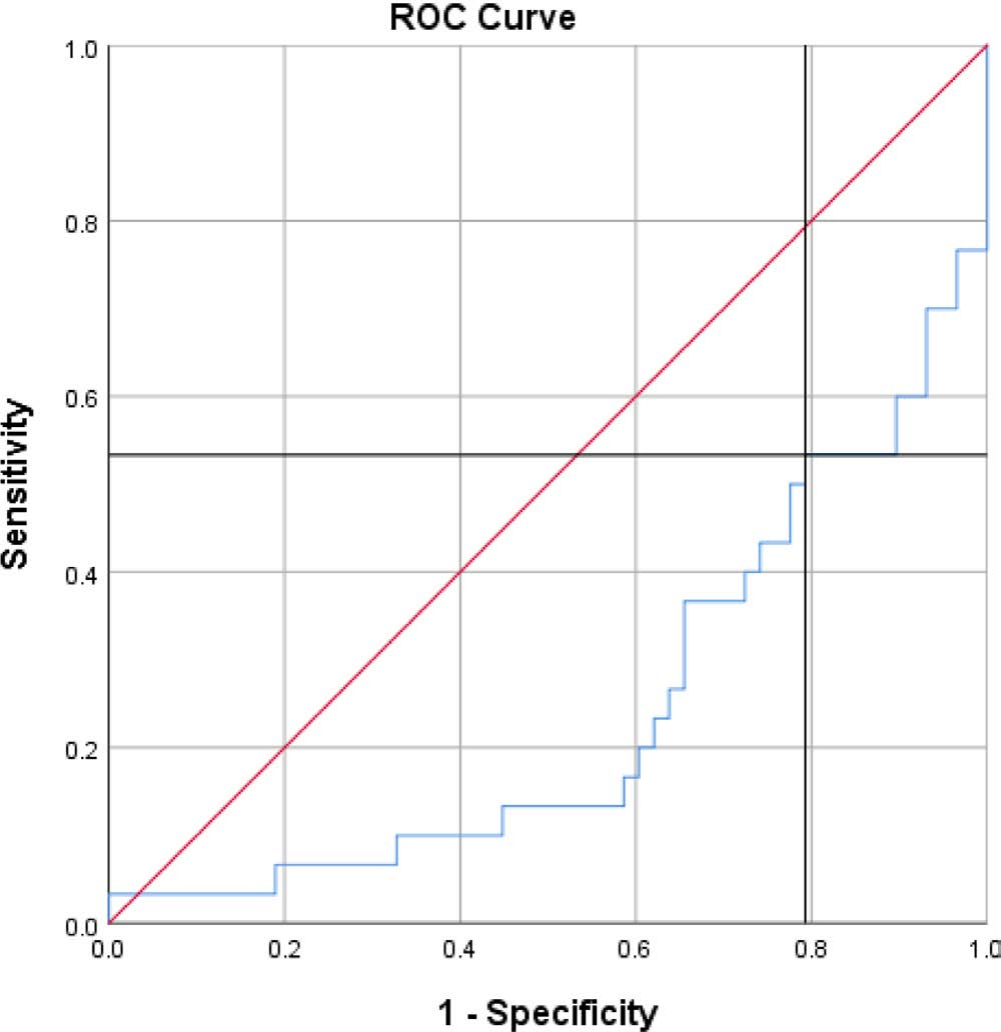
ROC curve for differentiation between STEMI and healthy, AUC of 24.3% and a *P* value of 0 (sensitivity = 53.3%, specificity = 20.7%). AUC area under curve, ROC = receiver operating characteristic, STEMI = ST-elevation myocardial infarction.

**Figure 3.**
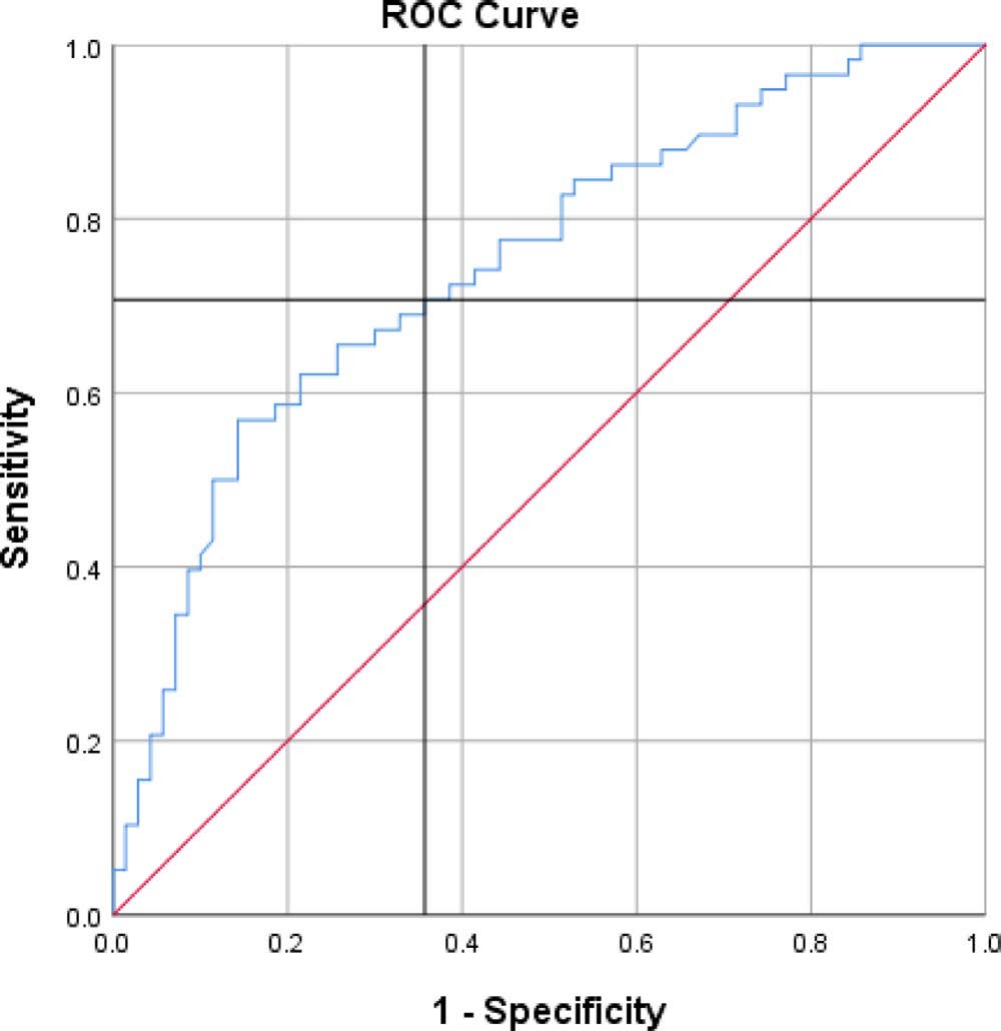
ROC curve for differentiation between NSTEMI and STEMI, AUC = 74.7%, *P* value = 0 (sensitivity = 70.7%, specificity = 64.3%). AUC = area under curve, NSTEMI = non-ST-elevation myocardial infarction, ROC = receiver operating characteristic, STEMI = ST-elevation myocardial infarction.

Our results showed that sCD40L is a statistically significant predictor of ACS, as indicated by a ROC curve with an AUC of 63.2% and a *P* value of .025 (Figures 1–3). A cutoff point of **5.680** was determined, with individuals above this value being more likely to have ACS, while those below were more likely to be healthy (sensitivity = 82%, specificity = 40%).

In the case of STEMI, a separate ROC curve was established for sCD40L, which showed an AUC of 24.3% and a *P* value of 0. A cutoff point of **14.350** was identified, with individuals above this value being more likely to have STEMI, while those below were more likely to be healthy (sensitivity = 53.3%, specificity = 20.7%). However, the ROC curves for sCD40L in differentiating NSTEMI and UA from healthy individuals were not statistically significant, with *P* values >.05.

As mentioned before, our study demonstrated a statistical difference in sCD40L levels between STEMI group and NSTEMI/Unstable groups. When combining NSTEMI and UA in 1 group and compared it to STEMI. A cutoff point of **22.600** was determined (AUC = 74.7%, *P* value = 0), with individuals above this value were more likely to have STEMI while those below this were more likely to have NSTEMI/UA (sensitivity = 70.7%, specificity = 64.3%).

## 4. Discussion

In this prospective case-control study that included 128 patients and 30 healthy individuals, we found that soluble CD40 ligand plays an important role in differentiating between ACS cases from healthy controls. Besides, our results showed that sCD40L was significantly higher in STEMI group among all of other ACS sub-groups and has a good ability to differentiate between STEMI patients from the healthy. In addition, when comparing between ACS subgroups, sCD40L could significantly differentiate between STEMI groups from NSTEMI/US group.

Soluble CD40 ligand (sCD40L) is a mediator with pro-inflammatory properties that is primarily released into the bloodstream by activated platelets. It interacts with various cells involved in vascular function, such as endothelial cells, macrophages, and T cells, and plays a role in the fundamental mechanisms of inflammation, atherosclerosis, and thrombosis.^[2]^ In terms of atherosclerosis and acute coronary syndrome (ACS), sCD40L has been found to contribute to the development, instability, and rupture of atherosclerotic plaques through different means. These include the infiltration of immune cells and the generation of inflammatory responses, which lead to an increased release of inflammatory cytokines.^[2]^ As a result, multiple studies have demonstrated that elevated levels of sCD40L can serve as indicators of increased risk for major complications or death in patients with ACS or chronic coronary syndrome (CCS).^[1–7]^ For instance, in a small case-control study involving apparently healthy women, higher levels of sCD40L were associated with an elevated risk of experiencing a combination of cardiovascular events.^[5]^ Another case-control study, which focused on 190 ACS patients, found that increased levels of sCD40L independently predicted death and recurrent heart attacks.^[7]^

In our study, we have identified soluble CD40 ligand (sCD40L) as a potential predictor for acute coronary syndrome (ACS). By setting a threshold of 5.680 ng/mL, we can effectively distinguish between individuals at higher risk of developing ACS and those at lower risk, who are more likely to maintain good cardiovascular health. Notably, this threshold demonstrates a sensitivity of 82%. However, it is crucial to acknowledge that the relationship between sCD40L and ACS remains limited and controversial. Several studies have reported no correlation between sCD40L concentrations and cardiovascular events.^[8–12]^

In STEMI, the complete blockage of a coronary artery leads to severe oxygen deprivation (ischemia) and death of heart muscle cells (necrosis). This triggers an intense inflammatory response in the body. During STEMI, activated platelets in the clot release sCD40L, this protein is cleaved from the membrane-bound CD40 ligand on platelets. The sCD40L then binds to CD40 receptors present on various cells, such as monocytes, macrophages, endothelial cells, and vascular smooth muscle cells. This binding of sCD40L to CD40 receptors amplifies the inflammatory cascade, leading to enhanced production of inflammatory cytokines, chemokines, and adhesion molecules, further propagating inflammation and tissue damage. Thus, STEMI patients have significantly higher levels of sCD40L compared to NSTEMI/UA patients, reflecting the greater degree of inflammation and cardiac injury.^[1,13–17]^ Accordingly, measuring sCD40L can help identify high-risk STEMI subgroups who may benefit from more intensive treatment. In our study, we observed a statistically significant difference in the average levels of sCD40L among patients with STEMI of 68.48 ng compared to other subgroups and healthy individuals which remained below 30 ng.

In addition, we identified specific cutoff points to distinguish between different conditions. For individuals with a value above 14.350, the likelihood of having STEMI was higher, whereas those below this value were more likely to be in a healthy state. This cutoff point showed a sensitivity of 53.3% and specificity of 20.7%. Furthermore, a cutoff point of 22.600 was established, indicating that individuals above this value were more likely to have STEMI, while those below were more likely to have NSTEMI/UA. This cutoff point demonstrated a sensitivity of 70.7% and specificity of 64.3%. Differentiating between STEMI and NSTEMI/UA is crucial because STEMI is an extremely dangerous and life-threatening condition that requires immediate medical attention, particularly within the first few hours of the artery blockage. Early diagnosis and prompt reperfusion are the most effective approaches to limit myocardial ischemia and infarct size, thereby reducing the risk of post-STEMI complications and heart failure.^[18]^

Given that over 95% of soluble CD40 ligand (sCD40L) originates from platelets, it has been assumed that the level of sCD40L reflects platelet activation.^[1]^ Consequently, it is important to consider that the predictive ability of sCD40L in acute coronary syndrome (ACS) may be diminished by antiplatelet medications, as these drugs inhibit platelet aggregation and the release of sCD40L. For example, in the analysis of a randomized study involving 1088 ACS patients who were either administered abciximab or a placebo, elevated concentrations of sCD40L were indicative of a higher risk of mortality or nonfatal myocardial infarction in the placebo group but not in the abciximab group.^[1]^ Thus, the measurement of sCD40L may have limited utility for assessing risk in patients receiving potent antiplatelet therapy.

### 4.1. Strengths and limitations

The sample size in this study was relatively small, which may limit the generalizability of the findings. However, this study possesses notable strengths that contribute to the existing literature on sCD40L and ACS prognosis. As the first study of its kind conducted in the Arabic World, this research fills a crucial knowledge gap and lays the foundation for future investigations in the region. Besides, this study categorized participants into specific ACS subtypes (STEMI, NSTEMI, UA) and included a control group of healthy individuals. This detailed classification allows for a more comprehensive evaluation of the prognostic value of sCD40L across different ACS presentations.

## 5. Conclusion

In summary, this study adds to the current body of knowledge on sCD40L by presenting findings that demonstrate elevated levels of sCD40L in patients with ACS and propose its potential as a biomarker for predicting cardiovascular events. The discovery of sCD40L as a predictive factor holds promise for improving risk assessment and personalized treatment approaches for ACS patients. However, it is important to acknowledge that the prognostic value of sCD40L remains uncertain due to contradictory findings from previous research. Therefore, additional investigations and larger-scale studies are necessary to delve into the underlying mechanisms and validate the clinical significance of sCD40L in enhancing patient outcomes and guiding therapeutic interventions.

## Acknowledgments

We wish to show our appreciation to **Stemosis for Scientific Research**, a Syria-based scientific research youth NGO managed by **Nafiza Martini**, for the scientific environment they provided. Also, we would like to extend our thanks to Dr Firas Almhesen from Michigan University for his consultations. We thank Dr Mohamad Moamen Almouallem for his contribution in bibliography.

## Author contributions

**Conceptualization:** Reham Nofal, Nafiza Martini, Majd Hanna, Ahmad Rasheed Alsaadi, Tahani Ali.

**Data curation:** Reham Nofal, Imad-Addin Almasri.

**Formal analysis:** Reham Nofal, Imad-Addin Almasri.

**Investigation:** Reham Nofal, Nafiza Martini, Majd Hanna.

**Methodology:** Reham Nofal, Nafiza Martini, Majd Hanna, Imad-Addin Almasri.

**Supervision:** Reham Nofal, Ahmad Rasheed Alsaadi, Tahani Ali.

**Writing—original draft:** Reham Nofal, Nafiza Martini, Majd Hanna, Imad-Addin Almasri.

**Writing—review & editing:** Reham Nofal, Nafiza Martini, Majd Hanna, Imad-Addin Almasri, Ahmad Rasheed Alsaadi, Tahani Ali.
